# Consensual Non-Monogamy (CNM) – Considering Sexual Minorities and Moving Away from a Dyadic Conceptualization

**DOI:** 10.1080/19317611.2025.2576764

**Published:** 2025-10-24

**Authors:** Dora Morar-Bolba, Rainer Banse, Terri D. Conley

**Affiliations:** ^a^ Department of Psychology, University of Bonn, Bonn, Germany; ^b^ Department of Psychology, University of Michigan, Ann Arbor, MI, USA

**Keywords:** CNM, sexual minorities, relationship satisfaction, sexual satisfaction, jealousy

## Abstract

**Objectives:**

Despite the growing interest in consensual non-monogamy (CNM), gaps and inconsistencies remain in this field of research. In the present study, we aimed to replicate and expand upon prior research on differences in relationship outcomes across monogamous and CNM individuals in a German sample.

**Method:**

Heterosexual and LGBQ individuals in monogamous, open, polyamorous, and swinger relationships (N = 1623) completed online measures of relationship satisfaction and its proposed predictors, sexual satisfaction, and jealousy. One-way ANOVA were conducted to investigate group differences. Hierarchical linear regression analyses were conducted to test the predictors of relationship satisfaction.

**Results:**

Our findings revealed that relationship satisfaction differed based on relationship type only among LGBQ individuals. Individuals engaging in monogamy were slightly less satisfied than individuals engaging in polyamory. Need fulfillment and effective communication were significant predictors of relationship satisfaction. For heterosexual individuals, sexual satisfaction was lower in the monogamous group than in the CNM groups. Regardless of sexual orientation, the monogamous group reported fewer, but more emotionally troubling instances of jealousy than any of the CNM groups, yet lower levels of jealous cognitions than the open relationship group.

**Conclusions:**

Our study differs from past studies in that we used a non-hypothetical approach to measuring jealousy and measured relationship concepts at the level of the relationship constellation. These new contributions along with the inclusion of individuals practicing nonhierarchical CNM provide more differentiated insights into the actual experiences of CNM and monogamous individuals.

## Introduction

Monogamy, the practice of being sexually and romantically exclusive with one partner, is conceptualized, within societal ideals, legal policy and within psychological theory, as a cornerstone of relationship success (Jordan, 2019, p. 160; Ryan & Jetha, 2010). However, research has started to find that monogamy might not be a prerequisite for thriving romantic relationships. Despite its bad societal reputation, often presenting as mononormativity (Kean, 2025; Pieper & Bauer, 2014), consensual non-monogamy (CNM) – a relationship arrangement by which partners agree to having multiple concurrent intimate, sexual and/or romantic relationships – is starting to receive recognition as a viable alternative to monogamy (Balzarini & Muise, 2020; Brooks et al., 2022; Moors, 2017).

Numerous studies have shown that individuals in monogamous and CNM relationships do not differ in terms of relationship satisfaction, commitment, and love (see, e.g. Balzarini, Dharma, Kohut, et al., 2019; Conley et al., 2017; Cox et al., 2021; Garner et al., 2019; Rubel & Bogaert, 2015). In most studies, trust, intimacy, and sexual satisfaction have even been reported to be higher in CNM relationships compared to monogamous ones, while jealousy levels are lower (Conley et al., 2017; Conley & Piemonte, 2021; Mogilski et al., 2019; Morrison et al., 2013) – although diverging results have also been observed (Conley et al., 2018; Wood et al., 2018). Several theories such as self-expansion theory, investment theory, and the Suffocation Model of Marriage have been used as frameworks to compare the functioning of CNM relationships with that of monogamous ones (Conley et al., 2017; Finkel et al., 2014). In light of these theories, CNM has on the one hand been argued to help improve relationship functioning and offer solutions to the problems of unmet needs, a lack of excitement and investment in the relationship and the danger of attractive alternatives (Conley et al., 2018; Finkel et al., 2014). On the other hand, possible drawbacks, such as increased time and resource investment, more intense emotional highs and lows and the experience of stigma have also been discussed (Conley et al., 2017; Moors et al., 2021).

Despite mounting evidence that CNM relationship arrangements can be viable alternative ways of constructing a relationship, prejudice against them is still widespread (Grunt-Mejer & Łyś, 2022; Schechinger et al., 2018). The general public believe that CNM relationships are less satisfying, less committed, have higher levels of jealousy and are linked to poor sexual health (Conley et al., 2013). Individuals in CNM relationships also report clinical practitioners being uninformed and lacking training in dealing with CNM related issues or expressing disapproval about CNM relationships and even refusing to provide therapy because of the relationship arrangement (Campbell et al., 2024; Schechinger et al., 2018). Therefore, furthering the research on CNM and ultimately the public’s and the professionals’ understanding and support of CNM relationship outcomes is a worthwhile endeavor that can help individuals who choose these arrangements have satisfying and thriving relationships (Rodrigues et al., 2024).

Despite advances in understanding CNM, certain aspects of these relationships remain understudied. Firstly, CNM is an umbrella term and is used to refer to quite diverse relationship arrangements. This diversity is however not always taken into account. Open relationships, polyamory and swinging are three distinct types of CNM. Open relationships generally involve partners being romantically exclusive, but engaging in sexual relationships with people outside of the couple (Buunk, 1980; Knapp, 1975; Watson, 1981). In swinger relationships a couple usually engages in extradyadic sexual activities at designated events such as swinger parties, mostly together, while remaining romantically exclusive to one another (Denfeld, 1974; Gilmartin, 1974; Jenks, 1998). In polyamorous relationships committed sexual and romantic bonds with multiple partners are welcomed (Taormino, 2008). When considering the different types, researchers uncover worse outcomes for individuals in open relationship (Conley & Piemonte, 2021). This speaks to the importance of looking at different CNM types individually instead of regarding them as a uniform group.

Secondly, another defining feature of a CNM arrangement is structure or hierarchy within the partnership(s). Hierarchical forms of CNM are arrangements in which the relationship to one partner, often referred to as the “primary partner”, is prioritized in terms of resources and commitment, while other partners take on less significant roles (Flicker et al., 2021). This is, however, not reflective of all CNM arrangements (Arter & Bunge, 2025). More than half of the participants in a study by Flicker et al. (2021) indicated that they do not have one primary partner. These instead live out different relationship constellations such as triads or quads sharing co-parenting responsibilities or multiple equal-status relationships that are independent of each other (Balzarini, Dharma, Kohut, et al., 2019; Sheff, 2011). Despite the diversity, almost all studies on CNM relationship quality exclude participants that do not have a primary partner (for an exception see Flicker et al., 2021). This considerably limits the generalizability of the results.

The argument of past studies for only including CNM individuals who have a main partner is the thus increased comparability between CNM and monogamous individuals. Though we do understand this line of thought, we wonder whether that does justice to the experience of CNM. The defining factor of a CNM relationship is that more than two people can be involved. By only focusing on one dyad within a relationship constellation, previous results can only speculate how satisfied relationship partners are on the whole. Is it the case that a more satisfying relationship compensates for a less satisfying one? Are the effects cumulative or does the satisfaction and dissatisfaction of one relationship spill over onto others? As multi-partnered nonhierarchical relationships are the reality for many CNM individuals, we aim to change existing measures so as to be able to investigate the relationship variables not only within main partnerships, but at the level of relationship constellations.

Thirdly, most studies do not include sexual minorities or do not account for sexual orientation when investigating CNM relationship outcomes. There is some disagreement about demographic differences between samples of CNM and monogamous individuals concerning age, gender, and education level (Fairbrother et al., 2019; Haupert et al., 2017), however one difference is reliably found across studies: sexual minorities (i.e. lesbian, gay, and bisexual) more often have a desire for and engage in CNM than heterosexual participants (Haupert et al., 2017; Lehmiller, 2020: Rubin et al., 2014). Overlooking sexual orientation is, therefore, particularly limiting. Most studies either only look at certain groups, such as polyamorous bisexual women, gay men in open relationships, limit the scope to heterosexual individuals, or collapse across individuals of different sexual orientations.

Not only for reasons of representation, but also to ensure the generalizability of results, it is important to account for sexual orientation. Scholars find reason to believe that especially bisexual and pansexual individuals might gravitate toward CNM, because it could enable them to live out sexual and romantic attraction with individuals of different genders (Moss, 2012). The increased desire and engagement of sexual minorities in CNM could also be explained by their tendency to question and reject societal norms, especially concerning relationships (Lehmiller & Selterman, 2022). On the one hand, this might render sexual minorities better equipped to nurture CNM relationships and account for higher levels of relationship functioning and satisfaction (Moss, 2012). On the other hand, the stigma resulting from the intersection of multiple stigmatized identities (e.g. being a lesbian and a swinger) could take its toll on individual well-being and negatively impact relationship satisfaction (Moors et al., 2021; Witherspoon & Theodore, 2021). All in all, there is good reason to take into account sexual orientation when investigating CNM relationship satisfaction.

## The current research

In light of the above-mentioned limitations, we aim to critically review and expand on previous studies to bring more clarity and a more nuanced perspective on the topic of CNM relationship outcomes. The current study aims to:

- replicate the work of Conley et al. (2017, 2018) and Conley and Piemonte (2021), concerning relationship satisfaction and the factors that explain it, sexual satisfaction and jealousy across different relationships styles
- expand existing research by:
    ○ considering the type of CNM practiced
    ○ exploring novel influences on relationship satisfaction
    ○ including sexual minorities
    ○ including CNM individuals with and without main-partner constellations
    ○ using a German sample, thus complementing the findings of mostly North-American-based studies

### Relationship satisfaction

At first glance CNM individuals seem to be as satisfied with their relationships as monogamous individuals (Balzarini, Dharma, Kohut, et al., 2019; Conley et al., 2017; Garner et al., 2019). However, when considering the type of CNM relationship studies do show differences in relationship satisfaction. Individuals in open relationships scored lower than swingers and polyamorists (Conley & Piemonte, 2021). Individuals in open relationships also reported less satisfaction than monogamous individuals, while swingers reported similar levels and polyamorists report higher levels of satisfaction (Conley et al., 2017).

Based on previous findings we expected individuals in open relationships to be less satisfied than individuals in monogamous, polyamorous and swinger relationships. We also expected monogamous individuals to be less satisfied than individuals in polyamorous and swinger relationships. Sexual orientation in the context of relationship outcomes by relationship type was examined in an exploratory fashion, as the lack of previous research did not allow for the formulation of specific hypotheses.

### Mediators of relationship satisfaction

Conley and Piemonte (2021) investigated several factors that could explain the lower level of satisfaction of the individuals in open relationship. Findings show that they communicate less effectively, are more extrinsically motivated to enter CNM relationships (motivated by long-distance relationship with the primary partner or by sexual dissatisfaction in the primary relationship) and are less likely to know the partner(s) of their partner(s), i.e. their metamours than polyamorists and swingers. These factors significantly accounted for differences in the satisfaction level of CNM individuals. Moreover, effective communication, motivation and metamour familiarity mediated the relationship between relationship type and relationship satisfaction. These findings provided first insights into the factors that contribute to relationship satisfaction in the context of CNM. We sought to replicate these findings. We also wanted to consider other factors that have been discussed in the CNM literature but that have not been incorporated into empirical research.

Need fulfillment is one such factor (Finkel et al., 2014; Moors et al., 2017). The general tenor is that engaging in multiple concurrent relationships – or having the freedom to do so, enables individuals to develop a wider network of intimate relationships that can fulfill their needs beyond what one partner could afford (Anapol, 2010). Help with childcare, household chores for cohabiting partners, a sense of belonging and community, access to new activities, a sense of self-expansion, growth and authenticity are all things that CNM individuals mention as benefits of their relationship (Moors et al., 2017). Finkel et al. (2014) suggested that engaging in CNM is one way that could help partners fulfill their higher order psychological needs, needs that are often frustrated in a monogamous relationship. Despite the comprehensive theory that exists regarding need fulfillment (Conley & Moors, 2014; Finkel et al., 2014), these assumptions still remain to be tested. It could be that different types of CNM relationships fulfill these needs to a different extent. Open relationships, in which partners have sexual contacts, but usually do not develop romantic or long-lasting intimate bonds, might satisfy fewer needs than polyamorous relationships, for example, which enable both sexual encounters and emotional connections (Balzarini, Dharma, Muise, et al., 2019). This could be a factor contributing to differences in relationship satisfaction between the groups.

One factor that was already present in previous studies, but that will be expanded on in the replication is effective communication. Conley and Piemonte (2021) constructed a new scale to measure individuals’ ability to communicate effectively, based on clinical interventions aiming to improve relationship functioning by improving partner communication. This scale was constructed for the purpose of the study and has not been used in other studies, which limits the ability to compare the findings to other findings on effective communication and relationship satisfaction. It would be interesting to see if established communication measures that are predictive of relationship satisfaction among monogamous individuals are also useful for CNM individuals. This would help establish the extent to which existing findings on effective communication, stemming from research on monogamous relationships, are applicable to CNM relationships. One well known theory of effective communication in romantic relationships has identified four communication styles, also known as the Four Horsemen of the Apocalypse, that are particularly detrimental to the quality of the relationship: criticism, contempt, defensiveness, and stonewalling (Gottman, 1999). Therefore, we will complement the scale developed by Conley and Piemonte (2021) with items measuring the extent to which the Four Horsemen are present in partner communication and use the responses on this augmented scale to predict relationship satisfaction.

Similarly, the factor of metamour familiarity, present in the study by Conley and Piemonte (2021), will be expanded on. According to self-help literature, getting to know one’s metamour can take away anxiety and help forestall jealousy, thus positively impacting one’s relationship with one’s partner. This leads us to wonder what would happen when familiarity with the metamour is high, but so is dislike for the person. Is familiarity sufficient or is the quality of the relationship also relevant? According to recent research, not only knowing the metamour, but having positive regard for and trusting the metamour, as well as having a positive impression of their relationship with one’s partner might be linked to relationship satisfaction (Arter & Bunge, 2024; Flicker et al., 2022). A positive relationship to the metamour has been suggested to facilitate comparison, i.e. the positive emotions one feels about other intimate relationships of one’s own partner. Since comparison is positively associated with relationship satisfaction, perhaps unsurprisingly, having a positive relationship and not just being familiar with the metamour, might have even more impact on the level of relationship satisfaction in CNM relationship arrangements. Therefore, along with testing the participants’ familiarity with their metamour(s), we will also test the quality of the relationship with the metamour(s).

Based on previous studies and literature we expected effective communication, need satisfaction, motivation to enter a CNM relationship, and metamour relationship to significantly predict relationship satisfaction and to mediate the relationship between an open relationship style and relationship satisfaction. Whether need satisfaction is, as we would expect, lower for individuals in open relationships than for individuals in swinger and polyamorous relationships was also tested. Sexual orientation (heterosexual vs. LGBQ) was also included as a predictor in the analysis.

### Sexual satisfaction

When it comes to sexual satisfaction, research has shown divergent results. A quantitative study by Conley et al. (2018), comparing monogamous individuals to individuals engaging in the different types of CNM has found slightly higher levels of satisfaction in the CNM as compared to the monogamous sample. Comparing across CNM types, the swinger group was significantly more satisfied. This study, however, did not use a validated measure of sexual satisfaction, but instead employed a three-item scale designed for the purpose of the study. Moreover, only participants in heterosexual relationships that had a primary partner were included. Wood et al. (2018) found no differences between CNM and monogamous individuals regarding sexual satisfaction, again with the primary partner. They used the New Sexual Satisfaction Scale to measure sexual satisfaction. Though sexual minorities were included in the study, sexual orientation was not considered when comparing the groups. Mitchell et al. (2020) used the same measure and also found no differences between monogamous individuals and CNM individuals. In this study multi-partnered CNM individuals were also considered. Satisfaction with the primary partner and satisfaction with the secondary partner were measured and comparisons between the two revealed mostly no differences. These two studies did, however, not differentiate between different types of CNM relationships.

A limitation of the findings on sexual satisfaction and CNM is that, to our knowledge, no studies measured overall sexual satisfaction across partners for individuals in CNM constellations. Many studies only measured sexual satisfaction with the primary partner (e.g. Conley et al., 2018; Wood et al., 2018). The sexual benefits people report in CNM relationships, however, most likely do not surface when considering only interactions with the primary partner. Some of the benefits mentioned are living out certain experiences with one partner that another partner does not desire (Kimberly & Hans, 2017), getting to explore one’s sexuality more deeply, feeling excited and empowered relating sexually with new people (Ritchie & Barker, 2007). Indeed, sexual satisfaction with non-primary partners probably spills over into the primary relationship, as CNM individuals report extradyadic involvement having a positive effect on their sexual satisfaction in their primary relationship (Palson & Palson, 1972). However, this is most likely only one part of the picture. Studies that included a secondary partner (Mitchell et al., 2020) offer a more detailed perspective, however, they do not capture the way that integration between the relationships manifests and is reflected in the overall satisfaction of CNM individuals with multiple sexual partners. Based on previous studies we expected CNM individuals to be more satisfied than monogamous individuals, and swinger individuals to be more satisfied than individuals in open or polyamorous relationships.

### Jealousy

Matters of jealousy often come to mind where CNM relationships are concerned. The assumption is that jealousy is pre-programmed if one’s partner is involved with other people (Conley et al., 2013; Ritchie & Barker, 2006). Nevertheless, Conley et al. (2017) found that CNM individuals report experiencing less emotional distress in comparison to monogamous individuals when imagining their partner being emotionally or physically involved with other people. The open, swinger and polyamorous group each reported lower levels of emotional jealousy than the monogamous group. Rogalski et al. found the same pattern of results regarding the jealous affect of monogamous compared to CNM individuals (not differentiating by type of CNM relationship), but a reverse pattern concerning jealous cognitions. Cognitive jealousy is, unlike emotional jealousy, higher among CNM individuals than among monogamous ones. This type of jealousy refers to the presence of worries or thoughts of the partner being unfaithful. This might be a reflection of the fact that CNM individuals are more often confronted with or discuss and process the possibility of their partners’ extradyadic involvement (Mogilski et al., 2019). Regardless of the underlying causes, the conclusion of these studies would thus be that a) thoughts of the partners’ engagement with other people surface more often for CNM individuals, but b) they are less emotionally troubling than in monogamous relationships.

One caveat with interpreting the results regarding emotional jealousy is that what is being measured is not actual experienced jealousy, but anticipated jealousy that may or may not be actually felt in hypothetical scenarios that may or may not occur. Conley et al. used the Anticipated Jealousy Scale (Buunk, 1982) and Mogilski et al. (2019) used the emotional subscale of the Multidimensional Jealousy Scale, that require participants to imagine hypothetical scenarios of their partner interacting in a possibly romantic or sexual way with other people. Studies show that people cannot always accurately predict how they will feel (Buehler & McFarland, 2001; Gilbert & Wilson, 2007). Some people might report feeling quite jealous when asked hypothetical questions in a survey, but are in actuality simply not confronted with situations that could promote jealousy in their day-to-day lives. They would thus not actually experience these feelings of jealousy in their relationship. This might be especially relevant, because monogamous and CNM individuals most likely do differ in exactly this regard. CNM individuals are, through the nature of their relationship arrangement on average, probably more often confronted with their partners’ extradyadic engagement. If this were the case, then monogamous individuals might in actuality be protected against situations that could provoke jealousy by their relationship arrangement – an effect obscured by the hypothetical nature of the measures.

Instead of looking at the anticipated level of jealousy, the present study examined the actual jealousy experienced in the current relationship (emotional and cognitive). To do this, participants were not asked to imagine hypothetical situations of their partners’ involvement with others and anticipate their emotions, but instead were asked to report on the emotions they did experience when such situations occurred. Allowing people to report not experiencing certain situations and only asking them to rate their jealousy in situations they did experience allowed us to compare the emotional responses of the two groups in lived-through situations. Based on previous studies, emotional jealousy should be lower in the CNM group than in the monogamous group. The opposite could be expected of cognitive jealousy. Moreover, following from the definition of CNM, it would be reasonable to expect that CNM individuals more often experience situations in which their partner is involved with other people.

In summary, we expected the lowest relationship satisfaction in the open relationship group and the highest in the polyamorous group. Expected significant and positive predictors of relationship satisfaction are effective communication, need satisfaction, motivation to enter a CNM relationship, and metamour relationship. We expected CNM individuals to be more sexually satisfied than monogamous individuals, with swinger individuals being most satisfied. Lastly, we expected emotional jealousy to be lower in the CNM groups than the monogamous group, and cognitive jealousy to be higher in the CNM groups than the monogamous group. New insights are to be gained about the role of sexual orientation regarding the relationship outcomes of different relationship types.

## Methods

### Participants

For the original data and more information on the analyses, consult the Open Science Framework (OSF) project linked to this paper here. Participants (N = 1973) were recruited via online groups and community fora. The survey link was distributed in regional networking groups in different German cities and in CNM-focused discussion groups. Since the focus of the study was on CNM individuals of different sexual orientations, targeted sampling was necessary in order to achieve the large sample size required to investigate the research questions. Criteria for participating were current involvement in a romantic relationship, being 18 years old or older and speaking German. Participation ensued via an online survey via SosciSurvey and was rewarded with the chance at winning one of four 25-euro vouchers. Only participants who met the inclusion criteria and completed the relevant measures were included in the final sample. Monogamous individuals were asked about cheating behavior and non-consensual non-monogamist (N = 131) were excluded. After the exclusion of 20 multivariate outliers using Mahalanobis distance the final sample used for further analyses consisted of N = 1623 participants (Table 1).

### Measures and procedure

The participants were first asked to supply demographic information and then proceeded to answer the relationship questionnaire. To measure sexual orientation, participants were asked to indicate whether they identify as heterosexual, gay/lesbian, bisexual, pansexual or identify as having another sexual orientation. For the latter option they could type their sexual orientation in a text box in an open answer format. To assess the type of relationship participants were engaged in, they were first asked if they and their partner had agreed to be monogamous or had implied monogamy. Those who had not agreed to be monogamous were given three descriptions of CNM relationship arrangements, as in the study by Conley et al. (2017) and asked to choose the description that best reflected their current arrangement. According to the description selected, individuals were coded as either “swinger”, in an “open relationship” or “polyamorous”. Monogamous individuals then completed measures of relationship satisfaction, sexual satisfaction and jealousy, need fulfillment and effective communication. CNM individuals completed the same measures and additionally a measure of extrinsic motivation for engagement in CNM. CNM individuals who reported having a metamour (i.e. who indicated that their partner(s) had other partner(s) besides them) received three questions measuring their relationship to their metamours. All measures were administered in German using either existing translations or new translations and adaptations created for the purpose of this study. All scales were to be answered on a 7-point Likert scale (1=“not at all/not often at all/not satisfied at all” to 7=“very/very often/very satisfied”).

### Relationship satisfaction

To measure relationship satisfaction the Relationship Assessment Scale (RAS) by Hendrick et al. (1998) was used in its German translation (Hassebrauck, 1991). As we decided against measuring the relationship variables only in regards to a main partner, the formulation of items had to be adapted for the CNM population to reference not satisfaction with one partner (“How well does your partner fulfil your needs”), but with their relationship constellation as a whole (“How well does your relationship constellation fulfil your needs”). Cronbach’s Alpha was 0.90 for the scale formulated for monogamous individuals and 0.80 when in the formulation adapted to CNM.

### Sexual satisfaction

A subset of seven items of the New Sexual Satisfaction Scale (NSSS) by Štulhofer et al. (2011) was used to assess sexual satisfaction with one’s sex life globally (as opposed to partner bound). Monogamous participants were asked to report how satisfied they were on a 7-point scale with their sex life in their current partnership, CNM participants were asked about their sex life in their current relationship constellation. Items that enquired about the satisfaction with a specific partner’s sexual behavior (assumed to also be the only partner, as the measure was developed for monogamous couples), were taken out. This only left items that could be answered when reporting on sex with one person as well as when reporting on sex with different people, such as “The frequency of my sexual activity”. These changes did not affect the reliability of the scales (Cronbach’s alpha = 0.90 for the monogamous version and 0.87 for CNM version).

### Jealousy

To assess jealousy, the subscales measuring emotional and cognitive jealousy of the Multidimensional Jealousy Scale, MJS (Pfeiffer & Wong, 1989) were used. As this instrument was conceptualized for heterosexual individuals, the items needed to be adapted for the purpose of this study (“X is flirting with someone of the opposite sex” became “X is flirting with someone”). Because of the considerable length of the study, two items with lower loadings were excluded from the scales (item 8 of the cognitive jealousy subscale and item 6 of the emotional jealousy subscale). This did not affect the reliability of the scales (Cronbach’s alpha 0.92 for the cognitive subscale and 0.91 for the emotional subscale in the monogamous version and 0.87 for both of the subscales for the CNM version).

### Need fulfillment

To measure the degree to which the satisfaction of higher order psychological needs relevant to the research question were met, participants completed the Need Satisfaction Scale (La Guardia et al., 2000) and the Self-Actualization Satisfaction Scale (Taormina & Gao, 2013). The first scale addresses the need for autonomy, for competence and for relatedness and consists of nine items. A short version of the Self-Actualization Satisfaction Scale consisting of six items (items 2, 3, 4, 8, 9, 11) was used. The items were also adjusted such that they specifically ask about need fulfillment in the context of the relationship (“In my relationship I am the person I always wanted to be.”, “Through my relationship, I finally fulfil all my innermost desires.”). The internal consistency of the new scale was high (Cronbach’s Alpha = 0.93).

### Effective communication

The Effective Relationship Communication Techniques Scale developed by Conley and Piemonte (2021) was employed. The Four Horsemen of the Apocalypse Questionnaire by Gottman (1999) was also used to measure effective communication. Two items for each of the four communication styles were included (except for “defensiveness”, for which only one item was added, since the scale of Conley and Piemonte (2021) already included one item measuring this style - item 11). The addition of these items (items 1, 8, 11, 12, 13, 22, 23) resulted in an 18-item scale. The internal consistency is high (Cronbach’s Alpha = 0.90).

### Relationship to metamours

CNM participants who reported having metamours (N = 283) were asked to answer three items relating to the metamour that plays the most significant role in their life (in case they had more than one). The first item was the one Conley and Piemonte (2021) used in their study to measure familiarity with the metamour. The other items measured the degree to which the participants liked the metamour and the degree to which the participants liked the relationship between their metamour and their partners. The internal consistency is high (Cronbach’s Alpha = 0.90).

### Extrinsic motivation

To measure the CNM participants’ motivation for engaging in their current relationship, the Extrinsic Motivations for CNM index by Conley and Piemonte (2021) was used. The scale consists of four items that provide possible extrinsic motivations for engaging in the relationship. High scores indicate high external motivation. A German translation of the scale was used. The answers on the four items were summed up instead of averaged, in order to capture the cumulative effects of different extrinsic motivators, as argued by Conley and Piemonte (2021).

## Analyses and results

One-way ANOVA were conducted to compare the means of the monogamous and the CNM group. For the first analysis the CNM group consisted of all CNM individuals collapsed across the three types of CNM relationship arrangements. For the second analysis, the comparison was done across the four relationship types: monogamous, open relationship, polyamorous and swinger. In case of significant results on this latter analysis, post-hoc Games Howell tests were performed in order to ascertain which group pairs show significant differences. Separate analyses were conducted for the heterosexual and the LGBQ individuals. This is a slight deviation from the preregistration, owing to the fact that we did not expect to be able to recruit enough LGBQ participants to analyze them as a separate group. In order to retain sufficient power, the LGBQ group was not further split into its subgroups.

Despite the ANOVA being robust against violations of its assumptions, non-parametric Kruskal-Wallis tests were also conducted for every hypothesis for which the assumptions of an ANOVA were not fully met. In most cases non-parametric tests yielded the same pattern of significance as the ANOVA. For those cases, only the ANOVA results will be presented as they ensure better comparability with results of previous studies. Concerning one hypothesis (the comparison of relationship satisfaction for LGBQ individuals in the monogamous and the CNM group), the ANOVA and Kruskal-Wallis test provided different results and therefore the results of both tests will be reported.

### Relationship satisfaction

For the heterosexual individuals, in line with previous studies, the ANOVA revealed no differences in relationship satisfaction between the monogamous and the CNM group, *F*(1, 691) = 0.93, *p* = .334. For the LGBQ individuals, as this study is first to show, the ANOVA revealed a small and just significant difference between the CNM and the monogamous group. The CNM group reported a higher level of satisfaction, *F*(1, 968) = 4.06, *p* = .044, η^2^ = 0.004. The non-parametric test however, revealed non-significant differences between the two groups (χ^2^ = 0.843, *df* = 1, *p* = .358).

**Figure 1.**
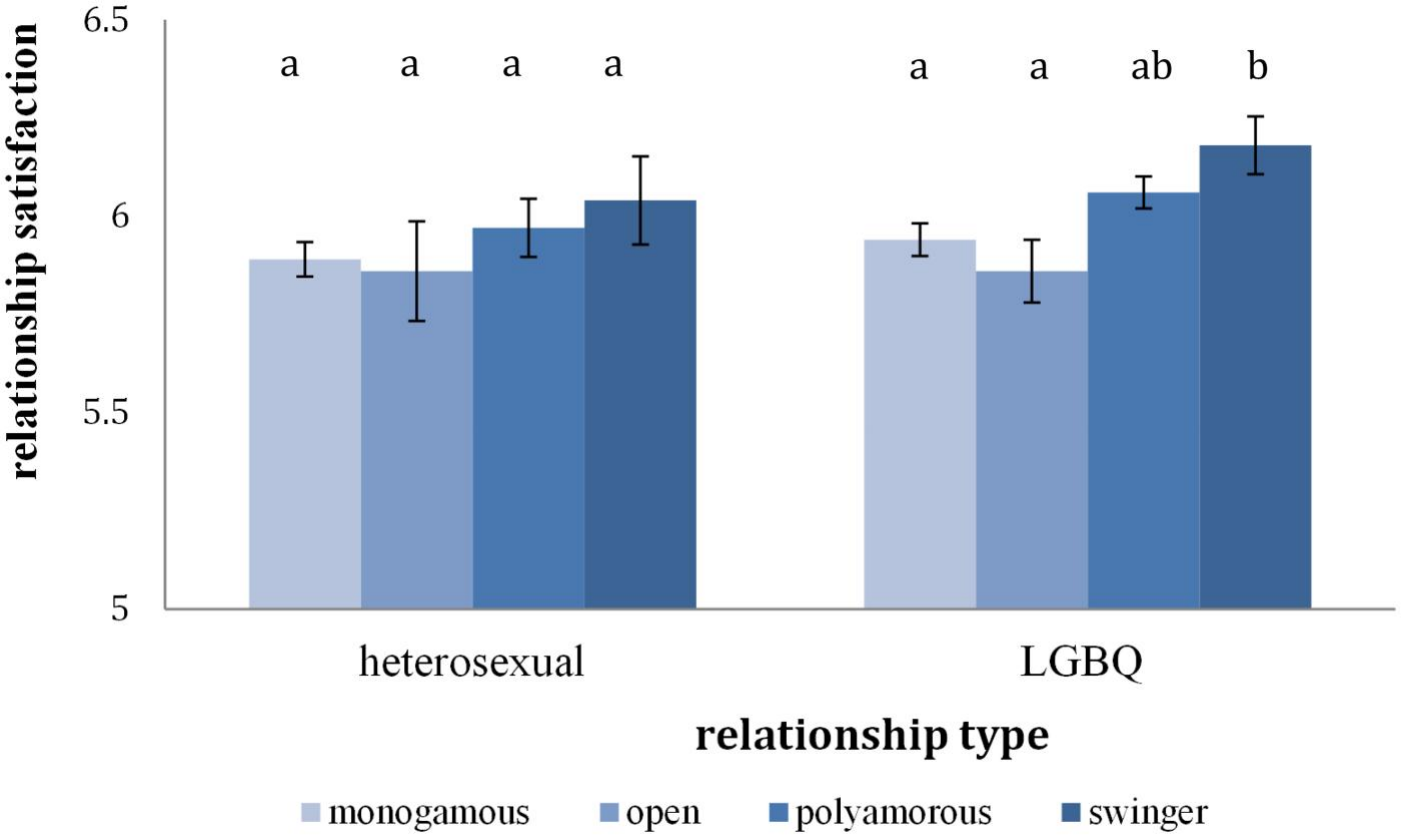
Relationship satisfaction across relationship type. *Note*. Bars that do not share the same index represent means that are statistically different (*p* < .05).

The second ANOVA comparing heterosexual individuals across the four groups (monogamous, open relationship, polyamorous and swinger) also found no significant differences, *F*(3, 689) = 0.65, *p* = .581 (Figure 1). This result was not in line with the expectations. When comparing LGBQ individuals across the four groups (monogamous, open relationship, polyamorous, swinger) the ANOVA revealed significant differences between at least two of the four relationship styles, *F*(3, 966) = 3.37, *p* < .05, η^2^ = 0.01 (Figure 1). Pairwise comparisons revealed that swinger individuals had higher levels of relationship satisfaction than individuals in open and monogamous relationships, but not than individuals in polyamorous relationships (Figure 1).

### Sexual satisfaction

For both the heterosexual and the LGBQ individuals the first ANOVAs revealed significant differences on sexual satisfaction between the monogamous and the CNM group. The CNM group had higher sexual satisfaction than the monogamous one, *F*(1, 659) = 35.10, *p* < .001, *η^2^*= 0.05 (heterosexual individuals) and *F*(1, 920) = 56.36, *p* < .001, *η^2^* = 0.06 (LGBTQ+ individuals).

**Figure 2.**
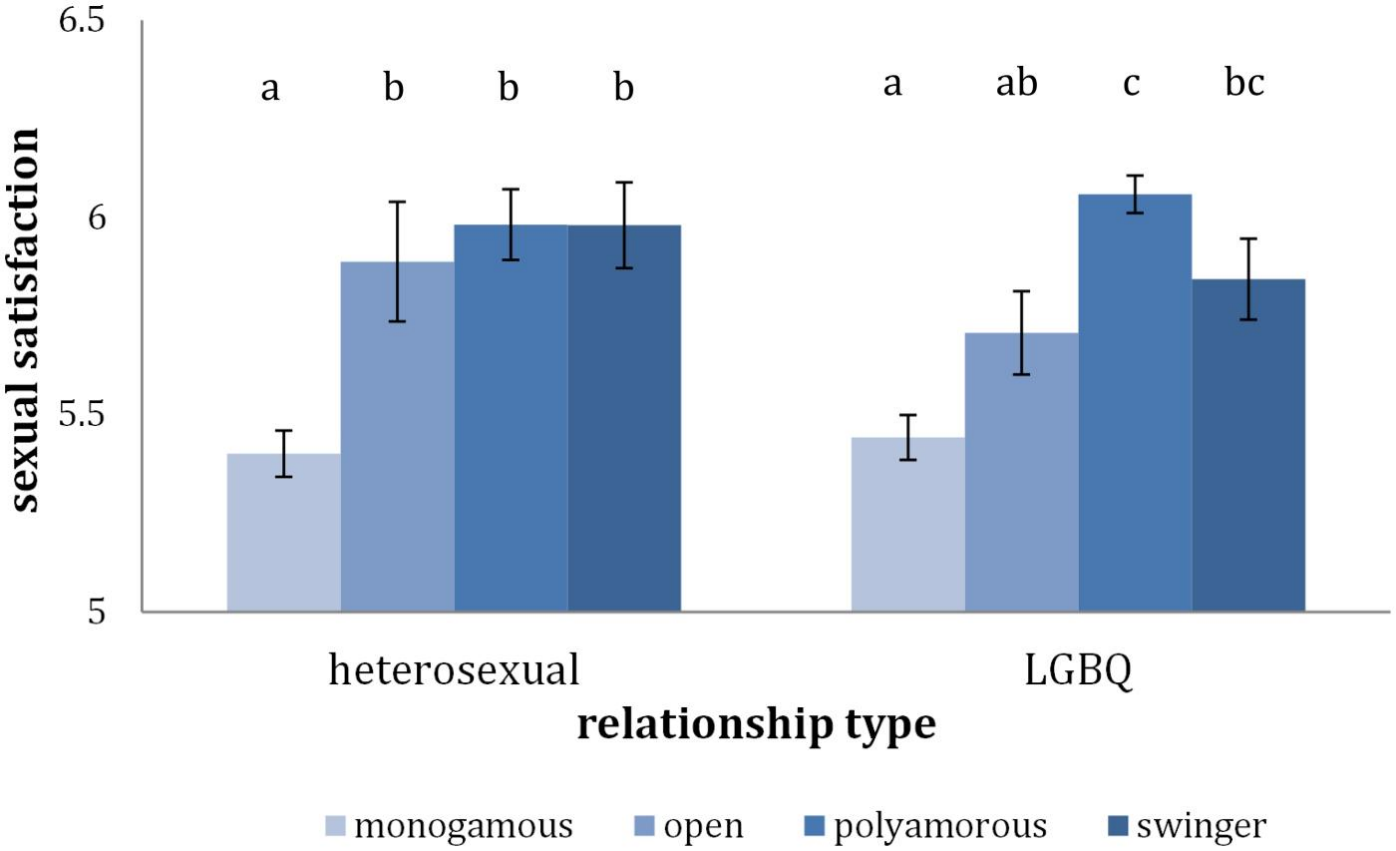
Sexual satisfaction across relationship type. *Note*. Bars that do not share the same index represent means that are statistically different (*p* < .05).

When comparing across all four groups (monogamous, open relationship, polyamorous, swinger) for both heterosexual, *F*(1, 657) = 11.76, *p* < .001, *η^2^* = 0.05 and LGBQ individuals, *F*(1, 918) = 21.85, *p* < .01, *η^2^* = 0.07 the ANOVAs revealed significant differences between at least two of the four relationship styles (Figure 2). Among the heterosexual individuals, pairwise comparisons revealed lower sexual satisfaction for the monogamous individuals than for the individuals in each of the three CNM groups. Among the LGBQ individuals, pairwise comparisons also revealed lower sexual satisfaction for the monogamous individuals than for the individuals in the swinger and polyamorous groups, but not lower than individuals in open relationships.

### Emotional jealousy

A considerable number of participants (67.21%) indicated not having experienced the situations described in the items, i.e. they did not experience their partner relating to other people in a potentially romantic or sexual way and were thus not able to rate the intensity of such experiences. Calculating the emotional jealousy mean score under these circumstances would not have yielded telling results. Instead, we calculated two scores and in doing so diverged from the preregistration. First, we calculated a frequency score based on the participants’ rating of whether they had experienced the situations described in the items in their current relationship. Second, we calculated an intensity score, employing the emotional intensity ratings of the participants that had experienced those situations.

**Figure 3.**
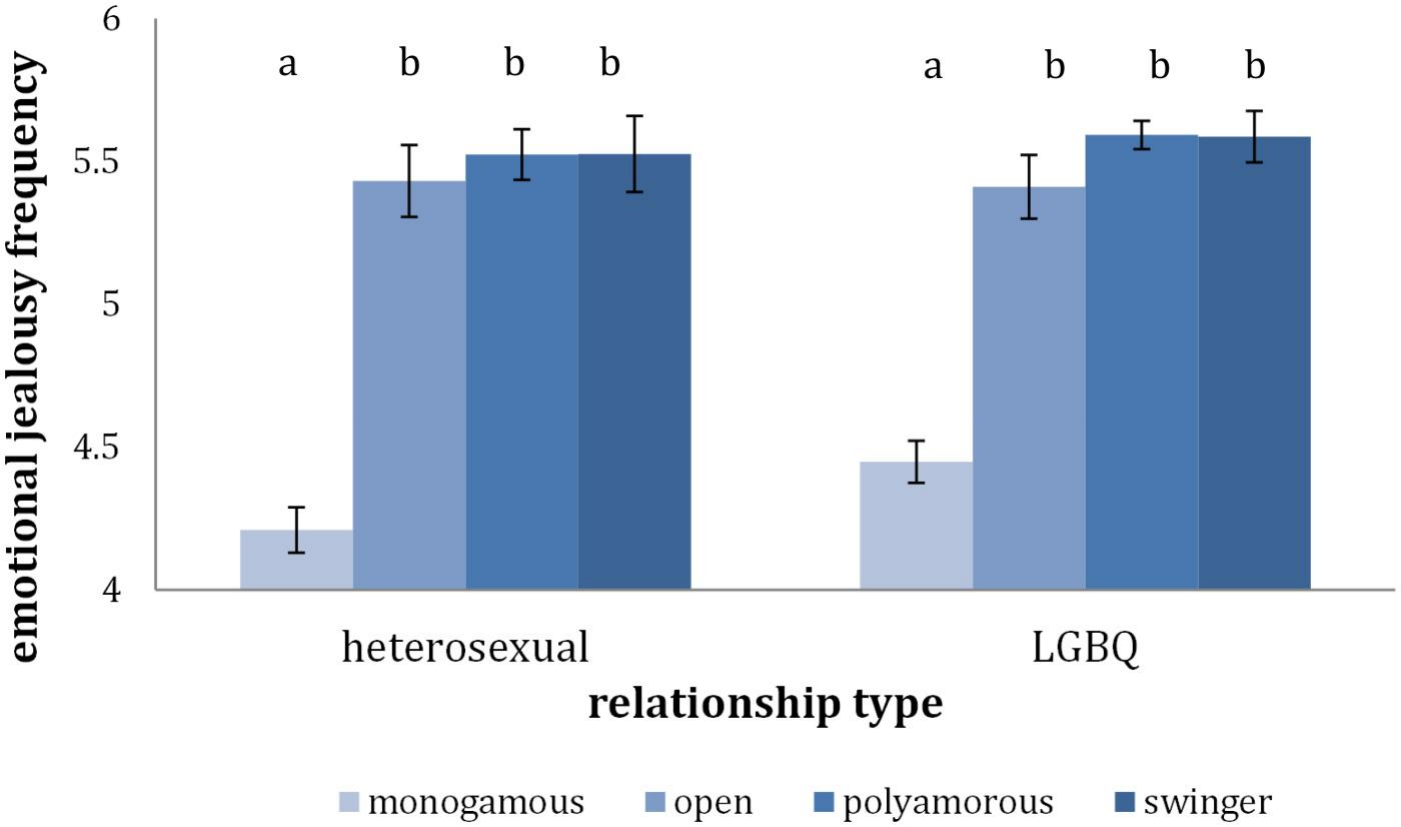
Frequency of situations of emotional jealousy across relationship type. *Note*. Bars that do not share the same index represent means that are statistically different (*p* <.05)

For both the heterosexual individuals and the LGBQ individuals analyses showed that the CNM group experienced situations of their partners involvement with or interest in other people significantly more often than the monogamous group, *F*(1, 745) = 111.78, *p* > .001, *η^2^* = 0.13 for the heterosexual individuals and *F*(1, 1024) = 179.55, *p* > .001, *η^2^* = 0.15 for the LGBQ individuals. For both the heterosexual and the LGBQ individuals each of the three CNM groups experienced significantly more such situations than the monogamous group, *F*(3, 743) = 37.22, *p* > .001, *η^2^* = 0.13 for the heterosexual individuals and *F*(3, 1022) = 60.30, *p* > .001, *η^2^* = 0.15 (Figure 3). These results were in line with our expectations.

**Figure 4.**
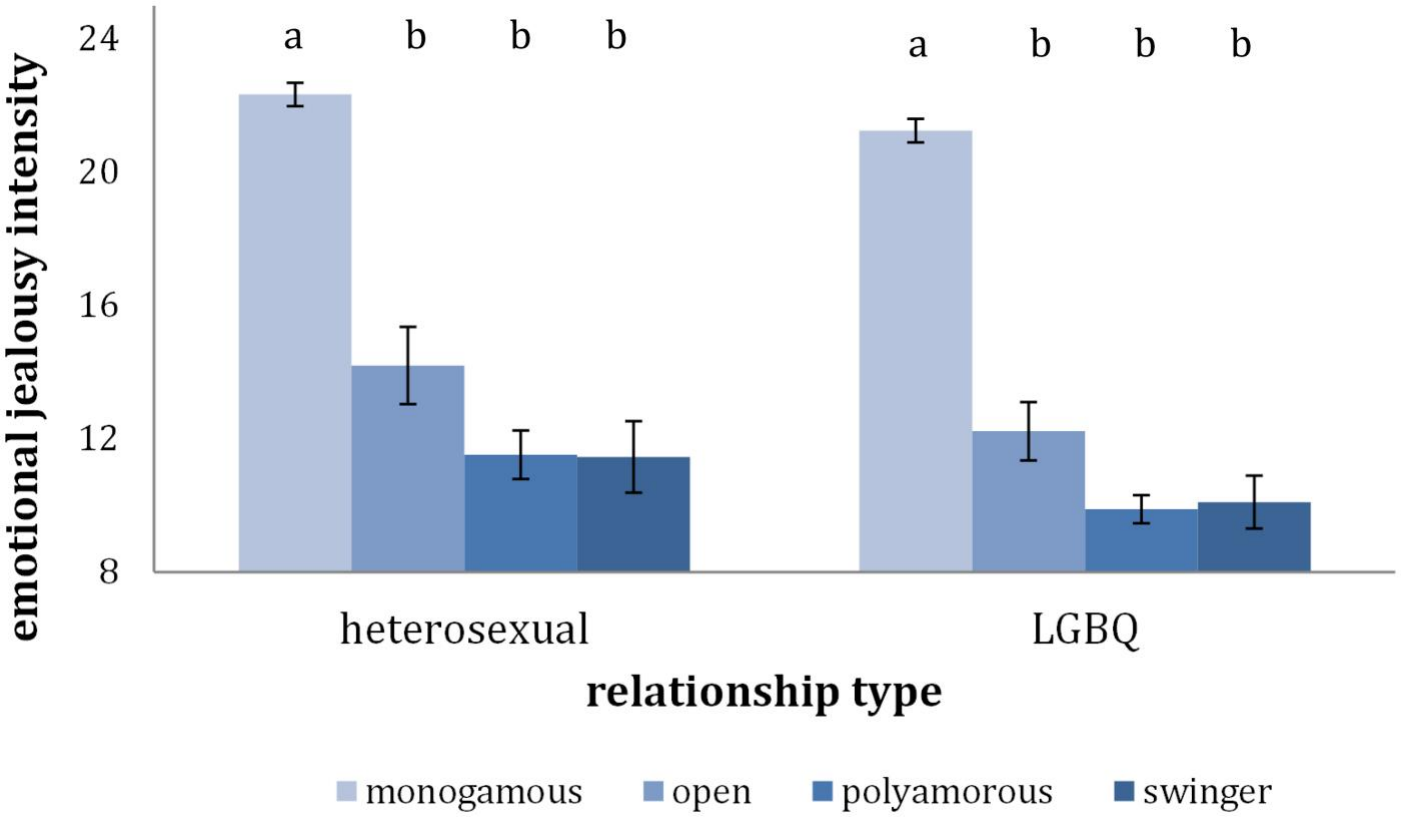
Intensity of emotional jealousy across relationship type. *Note*. Bars that do not share the same index represent means that are statistically different (*p* < .05).

Considering the intensity of the feeling, for both the heterosexual and the LGBQ individuals, the monogamous group reported experiencing significantly more negative emotions in these situations, *F*(1, 580) = 277.15, *p* < .001, *η^2^*= 0.32 for the heterosexual individuals and *F*(1, 845) = 483.27, *p* < .001, *η^2^*= 0.36 for the LGBQ individuals. For both the heterosexual and the LGBQ individuals the monogamous group experienced significantly more negative feelings than each of the three CNM groups, *F*(3, 578) = 94.61, *p* < .001, *η^2^* = 0.33 for the heterosexual individuals and *F*(3, 843) = 164.28, *p* < .001, *η^2^* = 0.37 for the LGBQ individuals (Figure 4). These results were in line with our expectations.

### Cognitive jealousy

For the heterosexual individuals the first ANOVA revealed significant differences between the monogamous and the CNM group on cognitive jealousy, *F*(1, 635) = 12.70, *p* < .001, *η^2^*= 0.02. The CNM group showed higher levels of cognitive jealousy than the monogamous group, which is in line with our hypothesis. For the LGBQ individuals there were no significant differences between the CNM and the monogamous group on cognitive jealousy *F*(1, 876) = 1.12, *p* = .29.

**Figure 5.**
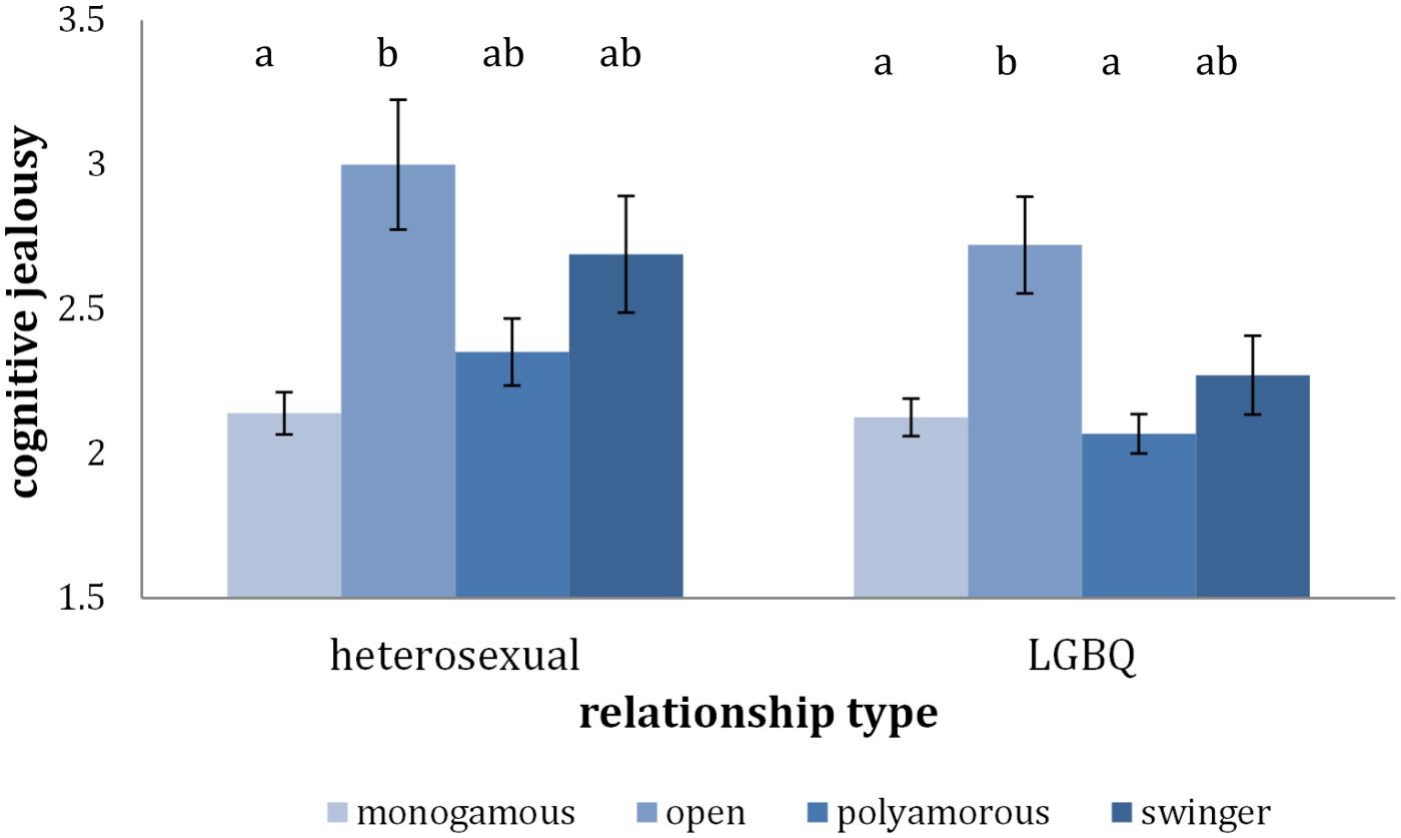
Cognitive jealousy across relationship type. *Note*. Bars that do not share the same index represent means that are statistically different (*p* <.05)

When comparing across all four groups, significant differences emerged between at least two of the four groups on sexual satisfaction for both the heterosexual *F*(3, 633) = 6.57, *p* < .005, *η^2^*= 0.03, and LGBQ individuals, *F*(3, 874) = 5.92, *p* < .005, *η^2^*= .02. For the heterosexual individuals pairwise comparisons revealed that the monogamous group had significantly lower levels of jealousy than the open group (Figure 5), while all the other group pairs did not significantly differ from each other. For the LGBQ individuals, the open group had significantly higher cognitive jealousy scores than the polyamorous and the monogamous group, but not than the swinger group (Figure 5).

### Mediators of relationship satisfaction

First, we tested whether communication, need fulfillment, motivation, and metamour relationship quality were lower for the individuals in open relationships than for those in the other two types of CNM relationships. Communication and need fulfillment were significantly lower, *F*(1, 633) = 13.10, *p* < .001, *η^2^* = 0.002 and *F*(1, 593) = 9.24, *p* < .05, *η^2^* = 0.02 Motivation and metamour relationship however were not significantly different between the open CNM type group and the other two CNM types group, *F*(1, 578) = 0.156, *p* = .70 and *F*(1, 271) = 2.70, *p* = .10. This was not in line with our expectations. To make sure this was not an effect of the change we made to the metamour factor we also ran the analysis with only the metamour item used by Conley and Piemonte (2021). The results of this analysis were however also non-significant, *F*(1, 271) = 3.31, *p* = .07.

Second, a Hierarchical Linear Regression Analysis was conducted to see if relationship type (open vs other type of CNM), effective communication, need fulfillment, metamour relationship quality, extrinsic motivation and sexual orientation predict relationship satisfaction. Relationship type alone was entered on the first step; relationship type along with need fulfillment, metamour relationship and motivation were entered on the second step of the analysis. On the third step, need fulfillment and sexual orientation were added along with all the other predictors (Table 2).

**Table 1.** Demographic characteristics.

Relationship type (n = 1623)	% Female	M (age)	% Heterosexual	% LGBQ	M (relationship duration)	% College educated
Monogamous (n = 852)	82	29 (18–77)	58.9	10.1 gay/lesbian 20.8 bisexual 8.9 pansexual 1.3 other	5.7	37.2
Open relationship (n = 140)	73.6	32 (18–64)	41.4%	7.9 gay/lesbian 37.9 bisexual 10.7 pansexual 2.1 other	3.1	40.7
Polyamorous (n = 501)	70.1	32 (18–65)	25.5%	4.2 gay/lesbian 36.7 bisexual 24.6 pansexual 9.0 other	3.0	49.9
Swinger (n = 130)	82.3	33 (20–52)	45.4%	2.3 gay/lesbian 40.0 bisexual 10.0 pansexual 2.3 other	4.5	35.4

Frequent entries specifying the other sexual orientations participants identify with were “asexual”, “demisexual”, “heteroflexible”, “bi-curious”.

The first model did not explain a statistically significant amount of variance on relationship satisfaction, and the standardized regression weight of the predictor relationship type was not significant. This was not in line with our expectations. The second model did explain a statistically significant amount of variance in relationship satisfaction. As for the individual predictors: the standardized regression weight for relationship type remained non-significant and the factor of extrinsic motivation was also not significant, diverging from previous results. In line with our expectations, communication and metamour relationship emerged as statistically significant predictors. More effective communication and a better relationship with one’s metamours predicted higher relationship satisfaction.

The third model was also significant and explained more variance than the second one. The factor of sexual orientation and the interaction between sexual orientation and relationship type were not significant. Need fulfillment reached significance as a predictor, while communication became non-significant. Need fulfillment and communication showed collinearity (*r* = .75). The metamour relationship factor was at the border of significance.

**Table 2.** Predictors of relationship satisfaction.

Step	Variable	*B*	*SE*	β	*p*	*F*	$R^{2}$	Adjusted $R^{2}$
1	CNM-Type (Open vs Other)	−.030	.176	−.010	.866	.029	.000	−.004
2	CNM-Type (Open vs Other)	−.055	.153	−.019	.722	23.56***	.260	.249
	Communication	.453	.053	.461	.000			
	Motivation	.004	.007	.029	.589			
	Metamour relationship	.094	.028	.176	.001			
3	CNM-Type (Open vs Other)	−.124	.125	−.043	.322	42.01***	.526	.513
	Communication	−.074	.061	−.075	.224			
	Motivation	−.005	.006	−.035	.424			
	Metamour relationship	.046	.023	.086	.051			
	Need fulfillment	.602	.050	.752	.000			
	Sexual orientation (Hetero vs LGBQ)	−.328	.482	−.207	.497			
	Sexual orientation*CNM type	.171	.248	.239	.492			

*** *p* < .001.

## Discussion

In summary, we could not replicate the result that individuals in open relationship were less satisfied than individuals in other CNM relationship types. Relationship satisfaction only differed based on relationship type for LGBQ individuals. LGBQ individuals engaging in CNM, particularly in polyamory, were slightly more satisfied than monogamous individuals. Need fulfillment and effective communication were significant predictors of relationship satisfaction. Sexual satisfaction for heterosexual individuals was higher in all CNM groups than in the monogamous group. Regardless of sexual orientation the monogamous group reported fewer, but more emotionally troubling instances of emotional jealousy than each of the CNM groups. Regardless of sexual orientation the open group reported more cognitive jealousy than the monogamous group. In general results did differ for the heterosexual and the LGBQ participants.

### Relationship satisfaction

In line with previous results, heterosexual individuals in monogamous and CNM relationships did not differ in their level of relationship satisfaction. However, diverging from results of previous studies, no significant differences concerning relationship satisfaction across the open, swinger and polyamorous groups emerged either. Though the open group did have a lower level of relationship satisfaction than the other two groups, as observed in the study by Conley and Piemonte (2021), this difference was not significant in the present study. Due to the fact that heterosexual and LGBQ individuals were analyzed separately, the group sizes of the groups used in this analysis were smaller than the ones used in the study to be replicated, despite the total sample size being equal. Nonetheless, there was still sufficient power to replicate small to medium results (power = 0.93). A possible explanation for the divergence is that this study measured relationship satisfaction at the level of the relationship constellation, while the original study measured relationship satisfaction with the primary partner. There is evidence that CNM individuals are more satisfied with their relationship to the primary partner than with their relationship to secondary partners (Balzarini, Dharma, Kohut, et al., 2019; Flicker et al., 2021). Thus, it is possible that when reporting on their relationship constellation their satisfaction was somewhat lower than when only reporting on their primary partnership.

As this study is first to show, slightly different results emerged when looking at LGBQ individuals. Among LGBQ individuals, the CNM group did report marginally higher relationship satisfaction than the monogamous group. Considering CNM-type differences, the open group did report lower relationship satisfaction compared to the polyamorous group, but not compared to the swinger group. The effect size for this difference was small. Several theoretical and methodical reasons for group differences between monogamous individuals and individuals in different types of CNM relationships have already been discussed (for an overview see Conley et al., 2017), however, it remains to be studied how sexual orientation plays into these differences. One theory might be particularly interesting when considering the relationship satisfaction of queer CNM individuals, namely social identity theory. It posits that people who experience connection and a sense of belonging stemming from their in-group identity, also experience higher relationship satisfaction (Kurdek, 2008; Mohr & Fassinger, 2006). In queer samples the social network formed around one’s identity is a factor that leads to partly higher relationship satisfaction than in heterosexual populations (Perales & Baxter, 2018). The same positive effect of belonging to a group because of one’s relationship identity has been theorized to be the case for CNM individuals, particularly for polyamorous and swinger individuals who have more opportunities to connect and find each other than individuals in open relationships (Conley & Piemonte, 2021). Perhaps the interplay of one’s queer identity and one’s polyamorous or swinger identity is particularly conducive to relationship satisfaction because of the opportunities for connection and belonging that it affords. This effect would be particularly pronounced in study participants since research on marginalized groups often rely on on- and offline communities to reach participants, research samples, ours included, consist of people who are part of communities. The impact of this feeling of belonging and community on the relationship satisfaction of CNM individuals is a topic worthy of further investigation.

### Mediators of relationship satisfaction

Diverging from the findings of Conley and Piemonte (2021), being in an open relationship did not predict lower relationship satisfaction than having a polyamorous or swinger relationship. Therefore, we cannot definitively say whether and how the proposed mediators contribute to explaining the relationship between CNM relationship type and relationship satisfaction.

The open group did communicate less effectively than the other two CNM groups and communication did play a significant role in explaining relationship satisfaction. These findings are in line with those of the study by Conley and Piemonte (2021). There was a slight nonsignificant trend toward the open group being less familiar with their metamours and relating less positively to them. Furthermore, the open group was not more extrinsically motivated to enter a CNM relationship and extrinsic motivation did not predict relationship satisfaction. A possible reason for the diverging results is that the items of the motivation scale were not as applicable to our sample. The scale names extrinsic motivators that are mainly only applicable to people who transitioned from a monogamous to a CNM relationship. Individuals who start out a relationship as CNM are not considered in the scale, which would also lead to the conclusion that the individuals that started out as CNM were less extrinsically motivated. In fact, the extrinsic reasons for choosing an open relationship arrangement from the beginning may simply not have been mentioned and the items mentioned were perhaps just not applicable for some. This is particularly problematic in the present study that also looks at nonhierarchical CNM in which likely more often than in hierarchical CNM a main dyad (potentially monogamous at first) never existed. Future studies could look at other reasons to engage in CNM from the start of a relationship, such as social pressure or social influence.

As for the newly-included variables, need fulfillment was significantly lower in the open group than in the other two CNM groups and was a significant predictor of relationship satisfaction. Need fulfillment correlated highly with effective communication and explained more variance than effective communication. Good communication is often argued to be necessary in CNM relationships in particular, because in these types of relationships partners need to be able to communicate their needs and their boundaries more actively and efficiently than in monogamous relationships. Thus, it is possible that effective communication contributes to relationship satisfaction in so far that it enables higher need fulfillment. Sexual orientation was also not significant and neither was the relationship between sexual orientation and relationship type, despite the findings presented above, which do point to differences between heterosexual and LGBQ individuals.

While it does seem that certain relationship-aiding traits are more pronounced in polyamorous and swinger relationships than in open relationships, the challenges are arguably different as well. Ultimately, the differences in relationship satisfaction are at most small. Effective communication is, for example higher in polyamorous and swinger relationships than in open relationships and is a factor that ultimately aids relationship satisfaction; however, it could be that it is also more necessary in the first two relationship types than in the latter where a don’t-ask-don’t tell policy is often preferred (Conley et al., 2017). Similarly, polyamorous and swinger relationships are associated with more need fulfillment than open relationships and need satisfaction contributes to relationship satisfaction, but the work that goes into the relationship is also arguably more intense as seen perhaps in the better communication that takes place. As Finkel et al. (2014) posited in the Suffocation Model of Marriage investing more in the relationship to be able to fulfill more needs is one strategy to increase relationship satisfaction, however not the only way. Expecting less from the relationship and looking for personal fulfillment outside of it is suggested as another viable option. Thus, different strategies of achieving relationship satisfaction may be employed in the different CNM relationship types and might lead to similar levels of relationship satisfaction, albeit in different ways.

### Sexual satisfaction

In the present study both for the heterosexual and the LGBQ individuals the CNM group reported significantly higher sexual satisfaction than the monogamous group. Noteworthy is the fact that this difference was quite large (η^2^ = 0.05 and η^2^ = 0.07, respectively). This is in contrast to previous studies that found a rather small difference, and other studies that found no difference at all (Conley et al., 2018; Wood et al., 2018). Methodologically the present study differs, in that participants were asked to not only report on sex with the primary and/or the secondary partner, but with all partners. Thus, more aspects contributing to sexual satisfaction than just those present in a primary partnership were accounted for. One such factor could be, as mentioned in the introduction, the possibility of experiencing and exploring different things sexually, than what is possible or desired in the primary partnership (Kimberly & Hans, 2017). Adaptations to the measurement instruments that allowed for this factor and probably others as well to flow into the assessment, by asking participants to consider all their current sexual partners, could explain the bigger group differences found in this study.

Sexual frequency might be another factor that could explain the bigger differences found. Sexual frequency in primary CNM partnerships did not differ from that within monogamous partnerships. However, as CNM participants could now report on sex with multiple partners, it is likely that the frequency they were reporting on was higher. As frequency is strongly correlated with satisfaction (McNulty et al., 2016), this could also help explain why the discrepancy was higher. If this is indeed accurate, it would speak for the importance of measuring satisfaction with all partners and not just with one or two. A defining feature of CNM relationships is that they enable individuals to relate to more people, if this leads to more frequent and more diverse sexual encounters, which leads to more satisfaction, then this is a relevant finding that reflects the reality of the different relationship types.

Among the heterosexual individuals the swinger, open and polyamorous groups were equally satisfied, while among the LGBQ individuals, the open group was less satisfied than the other two. Among the LGBQ individuals, the open group also reported lower relationship satisfaction, and as relationship satisfaction and sexual satisfaction are strongly positively correlated, the result is perhaps not surprising. One other difference to previous studies, is that the swinger group was not most sexually satisfied, but as satisfied as the polyamorous group. This might also have something to do with the adaptations in the measurement instruments that cued reporting satisfaction with all partners as opposed to satisfaction with only the primary partner. Swinger individuals generally have a main partner and usually engage sexually with others together with their main partner. This probably means that general sexual satisfaction is captured quite accurately when asking about satisfaction with the main partner as new sexual experiences are experienced together with the main partner. Polyamorous individuals may however have multiple independent relationships that share unique sexual dynamics which are only accounted for when asking about all partners. These findings clearly speak for the importance of accounting for sexual orientation and CNM relationship type.

### Jealousy

Among the heterosexual individuals, the CNM group reported more thoughts and worries about their partners’ engagement with other people than did monogamous individuals. This is in line with previous studies. Interestingly, though when taking a more nuanced approach and comparing across the CNM groups, only the open group scored significantly higher on cognitive jealousy than the monogamous group. For the LGBQ individuals there was no significant difference between the monogamous and CNM group on a whole, however the open group had significantly higher scores of cognitive jealousy than the monogamous and the polyamorous group. Grouping all types of CNM together might thus lead to a too coarse interpretation. Open relationships, in which partners often choose to not discuss their extradyadic engagement with each other, may leave more to worry about and be suspicious of. On the other hand, monogamous relationships, in which there is usually no reason to assume extradyadic engagement, and polyamorous and swinger relationships, where the engagement is more openly talked about seem to be associated with less cognitive jealousy.

Concerning emotional jealousy, the monogamous group reported significantly more negative feelings than the CNM group when encountering situations in which their partner is relating to other people. This finding is backed up by previous research and could provide support for the idea that CNM relationships might be attractive for people who do not struggle with jealousy as much in the first place, as well as the idea that CNM relationships are a training ground for dealing with jealousy (Conley et al., 2017; De Visser & McDonald, 2007). Following these results, it has often been argued that monogamy, despite common belief, does not shield one from jealousy, on the contrary monogamists would experience more jealousy. However, by asking participants to only report on their emotional response to actual experienced situations and not asking them to anticipate their response, the current study revealed that monogamous individuals experience far fewer situations in which their partners relate in a potentially sexual or romantic way with other people in the first place. Therefore, even if they feel worse when such situations happen, they experience these negative feelings of jealousy less often in their relationships because the relationship arrangement that they have chosen limits their exposure to such situations.

In conclusion, the relationship types investigated in this study might have different profiles that ensure satisfaction in different ways. In CNM relationships engagement with multiple partners would require partners to be able to discuss the relationship intensely and frequently, which goes hand in hand with worrying about a partner’s infidelity, however when done well this goes hand in hand with lower emotional distress and higher need fulfillment. Monogamous relationships on the other hand might have a lower potential for conflict, and thus a lower need for effective communication to keep the relationship thriving. Need fulfillment in the relationship may be lower, but perhaps more time and energy is spared and can be allocated to fulfilling endeavors outside of the relationship. Relationship characteristics such as the presence or absence of hierarchy and the sexual orientation of the partners remain understudied but relevant aspects.

Despite the new insights and the critical analysis of previous results there are some limitations to the study. Due to the characteristics of the sample required to answer the research questions, the random sampling of participants was not feasible. Also, the way in which cultural differences between the German and the mostly North-American samples used play a role in the difference in results is hard to pinpoint as the fundaments and the assumptions that the study relies on are based on the findings of studies on North-American samples, as little other research on the topic is available. Furthermore, though the study focused on another kind of diversity – namely that within relationship arrangements, it is a limitation that all non-heterosexual individuals were grouped together in the LGBQ group, not attending to possible differences among subgroups such as bisexuals and gay/lesbians.

Future studies could investigate to what extent and in what way specific sexual orientations interact with a CNM identity and reflect on satisfaction and other relationship outcomes. As this study is, to our knowledge, the first to conceptualize CNM relationship variables at the level of the relationship constellation and as the results indicate that this level of measurement does make a difference in the outcomes, future studies could look into the dynamics of relating intimately with multiple concurrent partners. This would be a necessary step in the direction of truly extending our understanding of relationships to include CNM experiences and move from understanding romantic relationships strictly at the dyad level to understanding the dynamics of more-person constellations.
